# Desempenho do teste rápido de chikungunya em cenário de cocirculação de Zika e dengue no Município do Rio de Janeiro, Brasil

**DOI:** 10.1590/0102-311XPT127424

**Published:** 2025-04-11

**Authors:** Geani de Oliveira Marins, Thiago de Oliveira Pires, Reinaldo Souza-Santos, Andréa Sobral, Rafael Freitas de Oliveira França, Elisa de Almeida Neves Azevedo, Marília de Albuquerque Sena, Raquel de Vasconcellos Carvalhaes de Oliveira, André Reynaldo Santos Perissé

**Affiliations:** 1 Escola Nacional de Saúde Pública Sergio Arouca, Fundação Oswaldo Cruz, Rio de Janeiro, Brasil.; 2 Centro Universitário Adventista de São Paulo, São Paulo, Brasil.; 3 Instituto Aggeu Magalhães, Fundação Oswaldo Cruz, Recife, Brasil.; 4 Instituto Nacional de Infectologia Evandro Chagas, Fundação Oswaldo Cruz, Rio de Janeiro, Brasil.

**Keywords:** Vírus Chikungunya, Testes de Neutralização, Curva ROC, Chikungunya Virus, Neutralization Tests, ROC Curve, Virus Chikungunya, Pruebas de Neutralización, Curva ROC

## Abstract

O Município do Rio de Janeiro é uma das cidades mais afetadas pela circulação simultânea dos vírus chikungunya (CHIKV), Zika (ZIKV) e dengue (DENV) no Brasil. Apesar de os testes rápidos estarem disponíveis comercialmente no país, ainda existem dúvidas sobre seu desempenho em cenários de cocirculação de flavivírus. O objetivo deste estudo foi avaliar o desempenho e melhor ponto de corte do teste rápido de CHIKV em cenário de cocirculação de Zika e dengue no Município do Rio de Janeiro. Foram incluídos 2.120 voluntários que residiam em domicílios particulares permanentes no Rio de Janeiro e que foram testados pelo teste rápido plataforma imunocromatográfica de duplo percurso (DPP, acrônimo em inglês) para CHIKV. Deste total, 769 participantes tiveram amostras de sangue venoso coletadas para confirmação diagnóstica pelo padrão ouro (teste de neutralização por redução de placas - PRNT) e testadas para CHIKV, DENV e ZIKV. Utilizou-se a curva ROC (*receiver operating characteristic*) para os cálculos de sensibilidade, especificidade, valores preditivos positivo e negativo e melhor ponto de corte. Das amostras analisadas, 15,5% foram identificadas com exposição prévia para CHIKV pelo teste rápido e 20,4% tiveram exposição prévia ao CHIKV pelo PRNT. Identificou-se exposição prévia para DENV em 89,2% das amostras testadas pelo PRNT e 67,8% para ZIKV. A sensibilidade e especificidade encontrada para o ponto de corte do fabricante foi de 96,1% e 97,5%, respectivamente. O melhor ponto de corte encontrado para o teste rápido foi de ≥ 14, para este resultado a acurácia foi de 97,7%, com especificidade e sensibilidade de 97,9% e 96,8%, respectivamente. Conclui-se que o teste rápido tem alto desempenho para detectar infecção por CHIKV em cenário de cocirculação de Zika e dengue.

## Abstract (en)

The municipality of Rio de Janeiro is one of the most affected by the simultaneous circulation of the chikungunya (CHIKV), the Zika (ZIKV) and the dengue viruses (DENV) in Brazil. Although rapid tests are commercially available in the country, there are still doubts about their efficacy in flavivirus cocirculation scenarios. This this study aimed to evaluate the performance and best cut-off point of CHIKV rapid tests in a scenario of cocirculation of Zika and dengue in the Rio de Janeiro. A total of 2,120 volunteers who lived in permanent private households in Rio de Janeiro were included and tested for CHIKV using the rapid test Dual Path Platform (DPP). Of the total number of participants, 769 had venous blood samples collected for diagnostic confirmation by the gold standard plaque reduction neutralization test (PRNT) and tested for CHIKV, DENV and ZIKV. The receiver operating characteristic (ROC) curve was used to calculate sensitivity, specificity, positive and negative predictive values, and the best cut-off point. A total of 15.5% of the samples were identified as having previous exposure to CHIKV according to the rapid test and 20.4% of the samples had previous exposure to CHIKV according to the PRNT. We identified prior exposure to DENV in 89.2% of the samples tested using PRNT and 67.8% to ZIKV. The sensitivity and specificity found for the manufacturer’s cut-off point was 96.1% and 97.5%, respectively. The best cut-off point found for the rapid test was ≥ 14, a result for which the accuracy was 97.7%, with specificity and sensitivity of 97.9% and 96.8%, respectively. It was concluded that the rapid test has high performance to detect CHIKV infection in a scenario of cocirculation of Zika and dengue.

## Resumen

El municipio de Río de Janeiro es una de las ciudades más afectadas por la circulación simultánea del virus chikungunya (CHIKV) y los virus Zika (ZIKV) y dengue (DENV) en Brasil. Aunque las pruebas rápidas están disponibles comercialmente en el país, todavía existen dudas sobre su realización en escenarios de cocirculación de flavivirus. El objetivo de este estudio fue evaluar el rendimiento y el mejor punto de corte de las pruebas rápidas de CHIKV en un escenario de cocirculación del Zika y dengue en el municipio de Río de Janeiro. Participaron 2.120 voluntarios que vivían en hogares privados permanentes en Río de Janeiro y que fueron evaluados por prueba rápida plataforma inmunocromatográfica de doble vía (DPP, por su sigla en inglés) para CHIKV. De este total, las muestras de sangre venosa de 769 participantes se recolectaron para realizar una confirmación diagnóstica mediante el estándar de oro (prueba de neutralización de reducción de placas -PRNT, por su sigla en inglés) y someterlos a pruebas de CHIKV, DENV y ZIKV. Se utilizó la curva de la característica operativa del receptor (ROC) para estimar la sensibilidad, la especificidad, los valores predictivos positivos y negativos, y el mejor punto de corte. El 15,5% de las muestras se identificaron con exposición previa al CHIKV por la prueba rápida; y el 20,4% de las muestras tenían exposición previa al CHIKV por PRNT. Se encontró una exposición previa a DENV en el 89,2% de las muestras evaluadas por PRNT y el 67,8% para ZIKV. La sensibilidad y la especificidad encontradas para el punto de corte del fabricante fueron el 96,1% y el 97,5%, respectivamente. El mejor punto de corte encontrado para prueba rápida fue ≥ 14, para este resultado la precisión fue del 97,7%, con especificidad y sensibilidad del 97,9% y del 96,8%, respectivamente. Se concluye que la prueba rápida tiene un alto rendimiento para detectar la infección por CHIKV en un escenario de cocirculación de Zika y dengue.

## Introdução

O vírus causador da chikungunya (CHIKV) foi identificado na região das Américas no ano de 2013 e se mantém como uma ameaça global pela natureza debilitante da doença ^1^. Os vetores *Aedes aegypti* e *Aedes albopictus* são responsáveis pela transmissão do CHIKV, sendo o *A. aegypti* o principal vetor e também transmissor dos vírus da dengue (DENV) e da Zika (ZIKV) ^2^. No Brasil, os primeiros casos de chikungunya foram oficialmente identificados em 2014, nos estados da Bahia e do Amapá, mas acredita-se que o vírus tenha circulado no país desde 2012, sem que houvesse detecção pelo sistema de vigilância ^3^. O CHIKV atingiu o sudeste do país em 2015, ano em que o primeiro caso de transmissão autóctone foi identificado no Estado do Rio de Janeiro ^4^. Atualmente, o Brasil apresenta o maior número de casos da doença nas Américas, com 7,4 casos/100 mil habitantes nas quatro primeiras semanas epidemiológicas de 2024 ^5^.

Com a introdução do ZIKV no Brasil em 2015 ^6^, o Município do Rio de Janeiro, capital do estado, enfrenta o desafio da cocirculação dos três arbovírus DENV, ZIKV e CHIKV, o que dificulta as ações para vigilância, controle e assistência de CHIKV ^7^. Nas 13 primeiras Semanas Epidemiológicas de 2024, foram notificados 644 casos de CHIKV no Município do Rio de Janeiro, uma incidência de 10,19 casos/100 mil habitantes ^8^, e nesse mesmo período foi decretada a epidemia de dengue no Rio de Janeiro ^9^. 

O cenário de cocirculação das três arboviroses é desafiador para o sistema de vigilância, pois os sintomas causados pela infecção do CHIKV podem ser confundidos com as infecções por DENV ou ZIKV, visto que essas arboviroses apresentam sinais e sintomas semelhantes e inespecíficos como febre, cefaleia, náuseas e vômito ^10^. Estes também são comuns a outras doenças febris, como por exemplo, influenza e malária ^11^. Assim, a notificação dos casos prováveis da infecção por CHIKV pelo sistema de vigilância do país é realizada por meio dos sintomas clínicos, e posteriormente os casos prováveis são confirmados ou descartados pelo diagnóstico laboratorial.

A infecção pelo CHIKV caracteriza-se por sintomas agudos e crônicos de natureza debilitantes como poliartralgias, que podem persistir por até 13 anos, afetando significativamente a qualidade de vida ^12^. As poliartralgias são o principal sintoma que difere a chikungunya da infecção causada pelo DENV. Outros desfechos foram relatados como complicações neurológicas, transmissão vertical e óbitos por descompensação de comorbidades preexistentes ^13^. Na fase aguda da doença, os indivíduos têm risco de óbito 8,4 vezes maior que indivíduos não infectados por CHIKV. Esses efeitos trouxeram a necessidade de maior demanda por serviços de saúde e aumento de recursos humanos e financeiros ^14^. 

Em áreas de circulação simultânea de Zika e dengue, o diagnóstico diferencial precoce de chikungunya é primordial, realizado principalmente pela técnica RT-PCR (*reverse-transcription polymerase chain reaction*), um método molecular caro e laborioso ^15^ indicado até o oitavo dia do aparecimento dos sintomas. A partir do quinto dia após o aparecimento dos sintomas recomenda-se a utilização de testes sorológicos. O teste de neutralização por redução de placas (PRNT, acrônimo em inglês) é um teste sorológico considerado padrão ouro na detecção de anticorpos neutralizantes para arboviroses, sendo relevante para regiões em que dois ou mais flavivírus ocorrem. O teste rápido para detecção de anticorpos IgM e/ou IgG é amplamente utilizado - o Brasil é o país com maior número de fabricação de teste rápido para CHIKV ^16^. Trata-se de um teste de triagem, de baixo custo, com simples execução, e que requer poucos recursos ^17^. Apesar dos benefícios, a acurácia do teste rápido de CHIKV ainda é desconhecida em cenário de cocirculação de arboviroses, em razão dos diferentes valores de sensibilidade e especificidade relatados nos estudos ^15^ ^,^ ^18^. 

No ano de 2018, foi realizado um inquérito populacional para arboviroses no Município do Rio de Janeiro ^19^ em que a prevalência populacional de chikungunya foi estimada em 18% com a utilização do teste rápido de CHIKV. No entanto, a acurácia do teste rápido em campo não era conhecida. Por essas razões, com base no inquérito soroepidemiológico, o objetivo deste estudo foi avaliar o desempenho e melhor ponto de corte do teste rápido de CHIKV em cenário de cocirculação de Zika e dengue no Município do Rio de Janeiro.

## Materiais e métodos

### Desenho, área de estudo e seleção dos participantes

Realizou-se um estudo de acurácia diagnóstica com base no inquérito epidemiológico de base domiciliar, com plano amostral de coleta por amostragem complexa do tipo conglomerado em dois estágios, realizado no período de julho a outubro de 2018, no Município do Rio de Janeiro - capital do Estado do Rio de Janeiro ^19^. 

O Município do Rio de Janeiro abrange uma área total de 1.255,3km^2^, distribuídos por 163 bairros distintos. Durante o período do inquérito epidemiológico, a população estimada do Município do Rio de Janeiro era de aproximadamente 6.688.927 habitantes. Caracterizado por um clima tropical quente e úmido, a temperatura média anual na cidade varia entre 20ºC e 30ºC, com períodos de chuvas abundantes especialmente no verão, conforme dados do Instituto Brasileiro de Geografia e Estatística (IBGE) ^20^. Além disso, a expectativa de vida no município é de 75,7 anos de acordo com informações da Secretaria Municipal de Governo e Integridade Pública ^21^. 

O estudo de Périssé et al. ^19^ recrutou voluntários aleatoriamente, com base nos setores censitários delineados pelo IBGE, conforme estabelecido na base geográfica operacional do *Censo Demográfico* de 2010 ^22^. Os setores foram ordenados de acordo com as Regiões Administrativas (RA). Uma equipe, composta por profissionais de saúde e entrevistadores, foi devidamente capacitada para assegurar a confiabilidade dos dados. Essa equipe conduziu entrevistas utilizando questionários eletrônicos e realizou testes laboratoriais, incluindo amostragem por punção digital (teste rápido) e coleta de sangue venoso para realização do teste de neutralização por redução de placas (PRNT - padrão ouro).

Neste estudo foram incluídos 2.120 voluntários que residiam em domicílios particulares permanentes no Rio de Janeiro e que foram testados com o teste rápido para as arboviroses estudadas no inquérito soroepidemiológico ^19^ no Município do Rio de Janeiro, sendo excluídos os participantes que não realizaram a coleta de amostra de sangue venoso para realização do PRNT.

A coleta do sangue venoso para confirmação por PRNT foi realizada em razão da cocirculação de mais de um flavivírus. Nesse caso, embora os testes rápidos apresentem alta acurácia em amostras laboratoriais, estes podem ser afetados pela reatividade cruzada entre DENV e ZIKV em campo. À vista disso, todas as amostras foram testadas para CHIKV, DENV e ZIKV.

Conforme descrito por Périssé et al. ^19^, as visitas domiciliares para coleta de amostras de sangue por punção digital, visando a realização do teste rápido de CHIKV, e coleta de amostras de sangue venoso para o PRNT, foram programadas para ocorrer nos finais de semana. Essa decisão foi tomada como estratégia para alcançar indivíduos que não puderam ser contatados durante a semana, seja por estarem ausentes no momento da entrevista ou por outros motivos. 

O material biológico foi transportado em caixa térmica com termômetro digital e gelo reutilizável entre 2ºC e 8ºC e armazenado em local de congelamento (-70ºC) na Fundação Oswaldo Cruz (FIOCRUZ). Posteriormente, as amostras de sangue venoso foram encaminhadas ao Laboratório de Virologia e Terapia Experimental do Instituto Aggeu Magalhães, FIOCRUZ Pernambuco.

As informações individuais e domiciliares coletadas por meio dos questionários padronizados podem ser consultadas no estudo publicado anteriormente ^19^.

### Tamanho amostral

Segundo Périssé et al. ^19^, a unidade primária utilizada para a seleção do estudo foram os setores censitários do Município do Rio Janeiro, conforme delineados pelo *Censo Demográfico de 2010* do IBGE ^22^. Esses setores foram inicialmente ordenados pelas RA e posteriormente pela renda média dos domicílios dentro de cada setor ^22^. Os domicílios foram considerados como unidades secundárias. A seleção dos setores foi realizada de acordo com as probabilidades proporcionais ao tamanho (PPT), em que o número de domicílios particulares em cada setor, conforme registrado no censo de 2010, foi utilizado como medida de tamanho. 

O tamanho da amostra foi calculado para estimar prevalências iguais ou maiores que 1,5% dos casos notificados para chikungunya. Especificou-se que o valor de margem de erro relativo (dR) deveria ser no máximo 35% (corresponde a um erro absoluto de 0,00525), com coeficiente de confiança (1 - α) de 95%. De acordo com a estimativa de 2,15 do efeito do plano amostral (EPA) ^23^, o tamanho da amostra calculado foi de 4.229. A amostra final foi composta por 2.120 moradores de 914 domicílios do Município do Rio de Janeiro. Os detalhes sobre a amostragem estão descritos em outra publicação ^19^. Para correção do viés de não-resposta utilizou-se modelagem das probabilidades de resposta com as variáveis de sexo e idade; para correção do viés nas estimativas utilizou-se peso amostral para cada participante.

### Procedimentos laboratoriais

A resposta imune ao CHIKV foi detectada por meio de kits de testes rápidos baseados na plataforma imunocromatográfica de duplo percurso (DPP, acrônimo em inglês), com detecção simultânea de IgM/IgG (Biomanguinhos, FIOCRUZ - Chembio Diagnostic System Inc.). Os resultados foram interpretados após cerca de 15 a 20 minutos, utilizando os pontos de corte estabelecidos pelo fabricante: CHIKV IgM/IgG - não reagente ≤ 16, indeterminado > 16 a < 20 e reagente ≥ 20; 

Utilizou-se o PRNT para avaliar a acurácia do teste rápido de CHIKV e confirmar a reação cruzada entre DENV e ZIKV. Os ensaios foram conduzidos em células Vero, em meio de cultura (Minimal Essential Medium, GIBCO), acrescido de 10% de soro fetal bovino (SFB), em microplacas de 24 poços (0,5mL/poço), preparadas 48 horas antes do ensaio. As amostras de soro foram inativadas (30 minutos a 56ºC) e diluídas (1/20, 1/80, 1/320 e 1/1280) em placas de microtitulação de 96 poços, e posteriormente incubadas com os vírus (DENV1 BR-PE/97-42735, DENV2 BR-PE/95-3808, DENV3 BR-PE/02-95016, ZIKV PE243 e CHIKV) a uma concentração contendo 30-70 UFP/mL. Após incubação (37ºC, 5% CO2) por 1 hora, as placas de 24 poços foram inoculadas, em duplicata, com 50μl de cada mistura vírus/soro, e incubadas (37ºC, 5% CO2) por 1 hora. Posteriormente, as células foram cobertas com 500μL de meio semissólido. Após incubação por seis dias para os flavivírus e dois dias para o CHIKV, as células foram fixadas com formalina a 3,5M, coradas com cristal violeta e posteriormente, contadas. 

A positividade foi definida com base na redução de > 50% na contagem de placas PRNT50 na menor diluição de soro utilizada (1/20). Os títulos de anticorpos para CHIKV foram estimados utilizando regressão não linear e transformados para escala logarítmica (log10) ^24^. O resultado negativo para as arboviroses foi definido pela formação de efeitos citopáticos do vírus, seguidos de alta formação de placas nas culturas e ausência de anticorpos no soro.

Foram considerados como casos de infecção neste estudo as amostras positivas para CHIKV pelo teste rápido (TR IgM/IgG) e confirmadas pelo teste padrão ouro (PRNT). 

### Análise estatística

Os dados de testes rápidos para CHIKV foram avaliados pelo método da curva *receiver operating characteristic* (ROC). Para encontrar o melhor ponto de corte da curva ROC, foi utilizado o índice de Youden (J), que sugere como melhor desempenho o que apresenta maior soma da sensibilidade e especificidade (sensibilidade + especificidade - 1), considerando o PRNT como padrão ouro. Os valores da estatística J, podem variar de 0 a 1; quanto mais próximo de 1 melhor o teste. Além disso, calculamos o valor preditivo positivo (VPP) e valor preditivo negativo (VPN) do teste rápido. Foram considerados o peso e plano amostral nas análises.

A descrição das estimativas de prevalência foi realizada por meio da distribuição da frequência absoluta e relativa, de acordo com o resultado do teste rápido para CHIKV (positivos e negativos) utilizando o ponto de corte estabelecido pelo fabricante (reagente ≥ 20) (Biomanguinhos, FIOCRUZ - Chembio Diagnostic System Inc.) e ponto de corte definido nesse estudo (reagente ≥ 14). As estimativas foram ponderadas pelo efeito do plano amostral e pesos amostrais utilizando o pacote estatístico *survey* ^25^. Foram fornecidos os intervalos de 95% de confiança (IC95%) para as estimativas.

As análises foram realizadas no programa R, versão 4.3.1 (http://www.r-project.org), utilizando os pacotes: *dplyr* ^26^, *srvyr* ^27^ *cutpointr* ^28^, *ROCR* ^29^, *plotROC* ^30^ e *MASS* ^26^.

### Aprovação ética

Esta pesquisa é uma continuação do *Projeto ZDC* que foi aprovado pelo Comitê de Ética em Pesquisa (CEP) da Escola Nacional de Saúde Pública Sergio Arouca (ENSP/FIOCRUZ; CAAE 83186318.1.0000.5240). Este estudo foi igualmente aprovado pelo CEP da ENSP/FIOCRUZ (CAAE 60624822.6.0000.5240).

## Resultados

**Figura 1:**
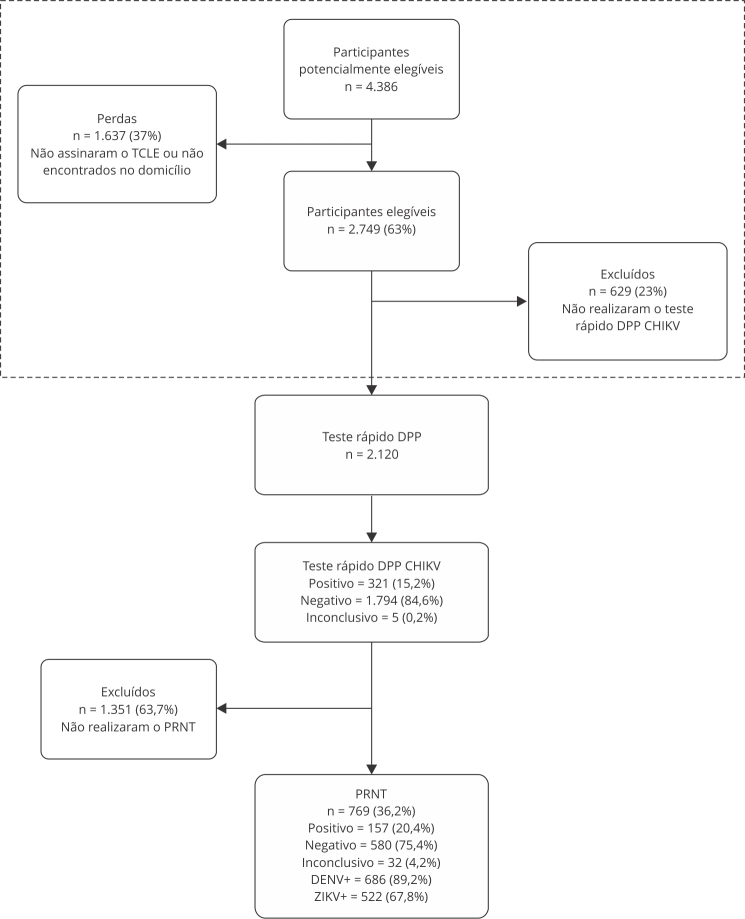
Fluxograma de seleção dos participantes do estudo.

CHIKV: vírus chikungunya; DENV +: positivos para vírus da dengue; DPP: teste rápido plataforma imunocromatográfica de duplo percurso; PRNT: teste de neutralização por redução de placas; TCLE: Termo de Consentimento Livre e Esclarecido; ZIKV+: positivos para Zika vírus. Nota: a linha pontilhada indica as etapas de seleção que foram realizadas no inquérito epidemiológico de Périssé et al. ^19^.

No inquérito soroepidemiológico realizado anteriormente ^19^, 4.386 pessoas foram consideradas potencialmente elegíveis. Contudo, após empregar os critérios de exclusão, 2.120 voluntários permaneceram no estudo e realizaram a coleta de amostra para o teste rápido. Neste estudo analisamos as amostras dos participantes que realizaram o teste rápido de CHIKV e identificamos que cerca de 15% destes voluntários foram positivos para CHIKV e aproximadamente 85% apresentaram sorologia negativa para CHIKV (Figura 1). Foram excluídos os indivíduos que não desejaram realizar a coleta da amostra de sangue venoso para a realização do PRNT (padrão ouro), que corresponde a 63,7% dos voluntários submetidos ao teste rápido. De acordo com a confirmação sorológica das amostras biológicas submetidas ao PRNT (padrão ouro), 75,4% das amostras testadas não apresentavam contato prévio de infecção por CHIKV, e 20,4% das amostras foram positivas para o vírus (Figura 1). Identificamos exposição prévia para DENV em 89,2% (n = 686) das amostras testadas pelo PRNT e 67,8% (n = 522) das amostras foram expostas ao ZIKV. 

Inicialmente, usamos os valores de titulação do teste rápido de CHIKV, estabelecendo o ponto de corte especificado pelo fabricante (reagente ≥ 20). Nesse cenário, obtivemos uma sensibilidade de 96,1% e especificidade de 97,5% para o teste rápido.

**Figura 2:**
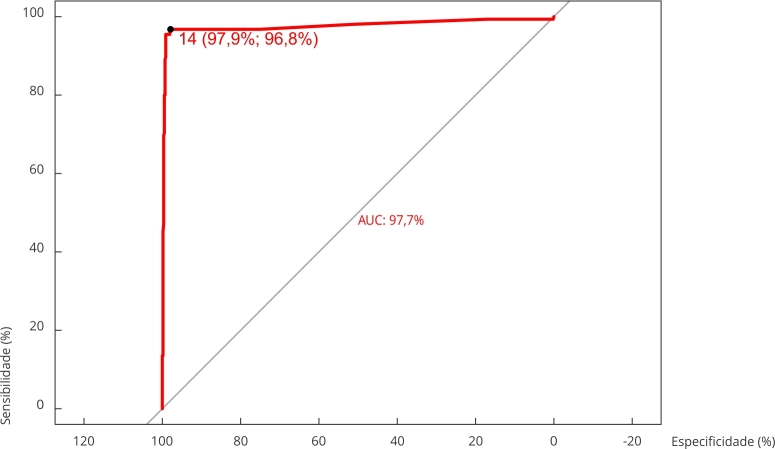
Determinação da acurácia e valor de corte para teste rápido anti-CHIKV (IgG) frente ao teste de neutralização por redução de placas (PRNT). Município do Rio de Janeiro, Brasil.

Posteriormente, avaliamos a acurácia do teste rápido e o melhor ponto de corte segundo a curva ROC. Observa-se na Figura 2, que a análise da curva ROC indicou o valor de corte de 14 para um ótimo desempenho do teste rápido, dado a área sob a curva de aproximadamente 98%. Desse modo, quando comparado com o PRNT, o teste rápido de CHIKV foi 96,8% sensível e 97,9% específico, com VPP de 92,6% e VPN de 99,1% (Figura 2). Nesta análise, o índice de Youden foi de 0,947.

AUC: área sob a curva. Notas: a linha vermelha representa o ponto de corte do modelo. Ponto de corte: 14; especificidade: 97,9%; sensibilidade: 96,8%; índice de Youden = 0,947; valor preditivo positivo = 92,6%; valor preditivo negativo = 99,1%.

Na Tabela 1, observamos a estimativa da prevalência de chikungunya utilizando o ponto de corte do teste rápido dado pelo fabricante e o melhor ponto de corte dado pela curva ROC. De acordo com as estimativas, a prevalência de chikungunya diferiu menos de 1% entre os pontos de corte e ele foi observado para a estimativa de casos negativos de chikungunya (Tabela 1).

**Tabela 1:** Distribuição das estimativas de prevalência de chikungunya segundo o ponto de corte do teste rápido (N = 2.120). Município do Rio de Janeiro, Brasil.

Casos de chikungunya	Teste rápido (Reagente ≥ 20) *	Teste rápido (Reagente ≥ 14) **
Positivo		
n^a^(N^)^b^	321 (1.038.182)	333 (1.074.160)
% (IC95%)^b^	15,5 (12,6-18,4)	16,0 (15,4-24,4)
Negativo		
n^a^(N^)^b^	1.794 (5.653.315)	1.767(5.574.773)
% (IC95%)^b^	84,5 (81,6-87,3)	83,3 (75,5-84,5)

IC95%: intervalo de 95% de confiança; N^: estimativa populacional considerando peso e plano amostral.

Nota: ^a^ amostra; ^b^ estimativa considerando peso e plano amostral.

* Ponto de corte fornecido pelo fabricante;

** Ponto de corte definido neste estudo.

## Discussão

Neste estudo, avaliamos o desempenho do teste rápido IgG para CHIKV em relação ao PRNT (padrão ouro). Verificamos que o teste rápido de CHIKV tem elevada acurácia no Município do Rio de Janeiro, cidade brasileira em que circulam conjuntamente os flavivírus DENV e ZIKV desde 2015. Contudo, identificamos como melhor ponto de corte para o teste rápido a regra de decisão 6 pontos abaixo do fornecido pelo fabricante, com VPP e VPN acima de 90%. A comparação entre o ponto de corte do fabricante e o ponto de corte indicado pela curva ROC para o teste rápido, revelou uma diferença inferior a 1% na detecção de exposição prévia ao CHIKV. Estimamos uma exposição prévia de 15,5% para o CHIKV no Município do Rio de Janeiro pelo teste rápido.

O CHIKV compartilha o mesmo vetor das arboviroses DENV e ZIKV que circulam simultaneamente no Município do Rio de Janeiro - em nosso estudo a confirmação diagnóstica de CHIKV e a possível reação cruzada entre os flavivírus foi testada pelo PRNT. Em alguns casos, o diagnóstico clínico é dificultado pelos sintomas inespecíficos e similares ao DENV e ZIKV, necessitando de testes sorológicos acurados ^10^. Estudos envolvendo testes sorológicos para CHIKV aumentaram após o surto no Oceano Índico em 2004, mas a acurácia ainda é desconhecida devido aos diferentes valores de sensibilidade e especificidade relatados ^31^. Estudos realizados há cerca de dez anos, com amostras de regiões endêmicas e que atualmente apresentam cocirculação de arboviroses relatavam baixa acurácia do teste rápido de CHIKV ^32^. No entanto, observa-se nos últimos anos uma melhora da acurácia do teste rápido de CHIKV pela necessidade de testes rápidos mais precisos e confiáveis devido às epidemias de CHIKV e das arboviroses que circulam simultaneamente ^15^. Nossos resultados concordam com o estudo de revisão sistemática e metanálise de Andrew et al. ^15^ de que o teste rápido de CHIKV apresenta bom desempenho com sensibilidade e especificidade superiores a 95% em áreas de cocirculação de arboviroses como Índia, Caribe e Marselha, na França. Ademais, em nosso estudo, os valores preditivos positivo e negativo indicam a alta capacidade do teste rápido de CHIKV, em classificar corretamente indivíduos com e sem a doença, com o VPN próximo a 100%.

Em regiões onde CHIKV, DENV e ZIKV são endêmicos e circulam simultaneamente, a capacidade de testes de triagem em identificar corretamente indivíduos positivos e negativos para CHIKV é essencial. Os valores de VPP e VPN do nosso estudo são superiores aos valores preditivos (VPP 85,6%; VPN 67,8%) de triagem baseada em sintomas clínicos de chikungunya (artrite, fadiga, erupção cutânea e dor na articulação do tornozelo) em cenário de cocirculação de cidades colombianas ^33^. Nossos achados corroboram o estudo de metanálise de Andrew et al. ^15^ que avaliou nove testes comerciais, os mais promissores entre eles desenvolvidos na Alemanha, no Reino Unido e nos Estados Unidos.

No estudo realizado na Coreia do Sul a sensibilidade e especificidade do teste rápido de CHIKV alcançou 100% ^17^. No entanto, o estudo utilizou amostras de Zurique, Suíça, local em que não é relatada a cocirculação de arboviroses, e a infeção de CHIKV é identificada em residentes que realizaram viagens a países endêmicos. A sensibilidade e a especificidade de 100% também foram encontradas no estudo de validação do teste rápido multiplex para DENV e CHIKV com amostras de plasma humano da Colômbia ^34^. A Colômbia é um país de relação fronteiriça com o Brasil que teve os primeiros casos autóctones de CHIKV confirmados em 2014. Assim como no Município do Rio de Janeiro, as três arboviroses (DENV, ZIKV e CHIKV) circulam na Colômbia desde 2015 e ambas as regiões apresentam algumas similaridades como aumento da densidade populacional, com áreas urbanas de baixo nível socioeconômico ^35^. 

O Município do Rio de Janeiro é caracterizado pelo rápido crescimento urbano com carências de infraestrutura, clima tropical quente e úmido com intensas chuvas no verão e áreas de baixo nível socioeconômico. Essas são algumas das condições que favorecem a proliferação do principal vetor (*A. aegypti*) do CHIKV, DENV e ZIKV no Município do Rio de Janeiro ^36^. Deste modo, a endemicidade de CHIKV e outros arbovírus são problemas epidemiológicos frequentes a serem enfrentados pelo sistema de vigilância no município. A utilização do teste rápido preciso auxilia no manejo adequado do paciente por ser um teste de fácil execução com resultado rápido ^18^. 

Anticorpos IgG anti-CHIKV podem ser detectados a partir do sexto dia e persistirem por anos ^12^. Assim, com a alta acurácia encontrada na detecção do teste rápido IgG anti-CHIKV nossos resultados estimam que 15,5% da população do Município do Rio de Janeiro foi exposta ao CHIKV. A soroprevalência encontrada em nosso estudo foi inferior ao inquérito realizado no Recife (Pernambuco), Nordeste brasileiro, no período de agosto de 2018 a fevereiro de 2019 ^37^. Esse estudo encontrou uma prevalência de 37% para CHIKV na cidade de Recife que assim como o Município do Rio de Janeiro enfrenta a cocirculação das arboviroses DENV, ZIKV e CHIKV em um contexto de desigualdades sociais e epidemias cíclicas de arboviroses. Entretanto, a Região Nordeste é a mais afetada pela epidemia de CHIKV no país ^38^.

Nossos achados foram próximos à prevalência de CHIKV relatada em contexto mundial, como demonstrado no estudo de revisão sistemática e metanálise de Zika, dengue e chikungunya, onde a soroprevalência de CHIKV foi estimada em 18% ^39^. Contrário ao nosso estudo, Skalinski et al. ^40^ encontrou prevalência geral de 24% para CHIKV, em uma revisão sistemática avaliando 71 inquéritos populacionais, sendo a maior parte realizada no Quênia (92,2%), no Brasil (9,4%) e na Polinésia Francesa (7,8%). 

A soroprevalência de CHIKV cresceu exponencialmente de 2017 a 2019 no Estado do Rio de Janeiro, com um grande surto de chikungunya no Estado entre 2018 e 2019 ^41^, seguido por um declínio dos casos com nova tendência de aumento em 2022. Nesse período, o Município do Rio de Janeiro apresentou maior prevalência em notificação dos casos em todos os anos comparado aos outros municípios do Estado do Rio de Janeiro ^42^. No ano de 2018, 10.746 casos de CHIKV foram notificados pela vigilância do Município do Rio de Janeiro. No entanto, considerando a soroprevalência estimada encontrada em nosso estudo, 1.038.182 casos deveriam ter sido notificados. As notificações do sistema de vigilância são referentes aos casos de indivíduos que procuram o serviço de saúde, geralmente casos sintomáticos, que correspondem a valores superiores a 70% nas infecções por CHIKV ^43^. 

Este estudo utilizou a base de dados de um inquérito epidemiológico realizado no Município do Rio de Janeiro para prevalência de DENV, ZIKV e CHIKV ^19^. Deste modo, participantes que não foram indicados para a coleta de sangue venoso e aqueles que não concordaram em realizar a amostra biológica para PRNT no estudo de Périssé et al. ^19^ foram excluídos. Apesar disso, corrigimos o possível viés utilizando peso amostral nas análises e incorporando o plano amostral. Ainda, a possibilidade de viés de seleção foi considerada no estudo de Périssé et al. ^19^ por meio de seleção dos domicílios de maneira aleatória de acordo com os setores censitários do IBGE e RA. Por se tratar de coleta de amostra biológica, crianças menores de cinco anos não foram contatadas para coleta de sangue venoso. 

## Conclusão

Nossos achados indicam que o teste rápido de CHIKV tem alto desempenho para detectar a infecção por CHIKV em área endêmica de Zika e dengue, apoiando as diretrizes de utilização do teste rápido para triagem diagnóstica em áreas de cocirculação de arboviroses. A redução do ponto de corte do teste rápido de CHIKV utilizado pelo fabricante pode aumentar minimamente a acurácia do teste rápido nesse contexto epidemiológico.
